# Evaluating the effectiveness of the “Double Reduction” education policy in China: A study using web scraping, sentiment analysis, and spatial regression

**DOI:** 10.1371/journal.pone.0335183

**Published:** 2025-11-03

**Authors:** Yan Xiao, Jinchen Xie

**Affiliations:** 1 Institute of Marxism, Xi’an Jiaotong University, Xi’an, Shaanxi, China; 2 Institute of Humanities and Social Sciences, Xi’an Jiaotong University, Xi’an, Shaanxi, China; 3 Department of Sociology, Northwest Agriculture & Forestry University, Xian Yang, Shaanxi, China; Shahjalal University of Science and Technology, BANGLADESH

## Abstract

**Background:**

The implementation of China’s “Double Reduction” (DR) policy, which aims to alleviate academic and extracurricular burdens, has received considerable attention. However, there has been limited evaluation of public satisfaction with the policy, particularly from a regional and multi-dimensional support perspective. This study aims to assess DR policy satisfaction from Chinese public, through a comprehensive “government–market–school” perspective.

**Methods:**

Combining the web scraping technology and sentiment analysis technology, this study captures 2,475,833 Weibo posts from 31 provinces in China related to DR policy. The causal relationship is discussed through spatial regression after controlling for spatial endogeneity.

**Results:**

The findings indicate that Chinese residents generally express positive satisfaction with the DR policy, however, substantial regional disparities persist. Provinces in the western and central regions exhibit lower increases in DR policy satisfaction (DRS) compared to those in the eastern region. All three dimensions—political, market, and educational support—have significant positive effects on DRS. Moreover, the results reveal positive moderations among the three types of support. Political support exerts a stronger influence on DRS in western provinces, whereas market support plays a more prominent role in eastern provinces. No significant interprovincial variation is observed for the effects of educational support.

**Conclusions:**

The study highlights the crucial role of political, market, and educational support in shaping public satisfaction with the DR policy. These findings suggest that targeted interventions are needed to address regional disparities, particularly in underdeveloped areas. Future research should focus on the long-term effects of the DR policy across diverse socio-economic contexts.

## 1. Introduction

Education has long been a cornerstone of social development and economic progress globally, with many countries striving to reduce excessive academic pressure and competition, particularly at the primary and secondary education levels [1,2]. Educational reforms aimed at reducing academic burdens have been implemented in several countries, yielding varying degrees of success. For instance, Finland’s educational reforms emphasize the reduction of students’ academic workloads, the promotion of self-directed learning, and the cultivation of comprehensive skill development, which has led to improved student well-being and learning outcomes [3]. Similarly, Japan and South Korea, despite persistently high levels of educational pressure, have gradually introduced measures to alleviate over-competition by implementing more flexible curricula, aimed at reducing academic anxiety and fostering students’ holistic development [4,5].

In China, rapid economic growth and the expansion of educational resources have led to intensified competition in primary and secondary education. The overdependence on private tutoring and exam-oriented instruction has worsened educational inequalities and adversely affected students’ physical and mental health. In response, the Chinese government introduced the “Double Reduction” policy (DR policy), which aims to alleviate students’ academic and extracurricular burdens, regulate the private tutoring sector, and reinforce the central role of school-based education, ultimately striving to achieve equitable and high-quality education nationwide [6].

The effectiveness of the DR policy remains insufficiently examined. Existing assessments primarily focus on macro-level outcomes, such as government performance, school quality, and student exam scores [7–9], while failing to incorporate a comprehensive evaluation from a “government–market–school” perspective. Such a multidimensional approach is essential, given that education operates as a semi-public good. Moreover, previous evaluations, often constrained by small sample sizes and limited data, may compromise the generalizability of their findings.

Accordingly, this study employs a combination of web scraping and sentiment analysis techniques, obtaining 2,475,833 Weibo posts related to the DR policy. Supported by spatial econometric modeling, the study addresses the following research questions: 1. What are public attitudes toward the DR policy in China? 2. How are these attitudes distributed across space, and which regions exhibit more favorable views? 3. What are the key determinants of public attitudes toward the DR policy in China?

## 2. Methods

### 2.1. Key techniques for obtaining the dependent variable

#### 2.1.1. Web scraping technology.

This study employs web scraping techniques to gather public sentiment and attitudes toward the DR policy from Weibo, one of China’s major social media platforms, to assess its regional implementation using big data. Web scraping—a method that connects user IP addresses with webpage URLs to automatically extract online content—has been increasingly adopted in social science research to analyze public opinion on social issues [10,11]. The procedure involves four key steps: (1) initializing the Uniform Resource Locator (URL), (2) queuing URL tasks, (3) matching user URLs with webpage ports, and (4) extracting webpage content into the processing pipeline. This approach yields 2,475,833 policy-related Weibo posts from 31 provincial-level administrative regions across China.

Given that Weibo data is unstructured—with irregular formatting, incomplete content, and no predefined schema—rigorous preprocessing is required to prepare it for empirical causal analysis. The preprocessing procedure comprises three key steps: (1) Deduplication, using R’s “unique” function to eliminate repeated posts from the same user; (2) Noise reduction, employing the “tm” package in R to remove stopwords and punctuation; and (3) Tokenization, utilizing Python’s “jieba” library to segment sentences into individual lexical units.

#### 2.1.2. Sentiment analysis technology.

Sentiment analysis, a core method in natural language processing (NLP), extracts the polarity and intensity of public attitudes from unstructured textual data, enabling systematic evaluation of sentiment on specific issues [10,11]. Two main approaches exist: machine learning-based and lexicon-based methods. The former requires extensive labeled data and computational resources, while the latter, as an unsupervised technique, utilizes predefined sentiment lexicons to identify emotional tone with greater efficiency and scalability. This study adopts the lexicon-based approach.

This study employs the NRC-D lexicon, developed by the National Research Council of Canada, which includes 14,183 emotion-labeled terms categorized into eight dimensions: Anger, Fear, Sadness, Disgust, Joy, Anticipation, Trust, and Surprise. Based on Plutchik’s emotional model, the first four are classified as negative emotions, while the latter four represent positive emotions [10,11]. The NRC-D lexicon is widely utilized in social science research for quantifying public sentiment.

Using web scraping and sentiment analysis, this study quantifies “DR Policy Satisfaction” (DRS)—the net change in positive sentiment during policy implementation—as the dependent variable at the provincial level.

### 2.2. Independent variable

This study evaluates the DR policy through a comprehensive “government-market-school” framework. Drawing on prior research [12–14], we construct thirteen secondary indicators grouped under three primary dimensions: political support, market support, and school support, to examine their influence on DRS (see Table 1).

**Table 1:** Indicator system of potential factors influencing the implementation of the double reduction policy in China^a^.

Primary indicators	Secondary indicators	Label	Description	Weights
Political support	Central fiscal support	θ_1_	Total central government budgetary allocation for basic education within the province ^b^	0.381
	Local fiscal support	θ_2_	Total local government budgetary allocation for basic education within the province ^b^	0.295
	Policy salience	θ_3_	Number of provincial-level policy documents related to the DR policy ^b^	0.230
Market support	Marketing education revenue	θ_4_	Total private-sector revenue from education within the province ^b^	0.333
	Marketing iInstitutions	θ_5_	Total number of market-based educational institutions within the province ^b^	0.733
	Marketing education employment	θ_6_	Number of employees in market-based educational institutions within the province ^b^	0.233
School support	Student-teacher ratio	θ_7_	Ratio of total number of students to total number of teachers in the province ^b^	0.589
	Cultural resource density	θ_8_	Total number of library holdings in educational institutions within the province ^b^	0.039
	Sport resource density	θ_9_	Total number of gymnasiums in educational institutions within the province ^b^	0.029
	ICT resource density	θ_10_	Total number of tablet computers in educational institutions within the province ^b^	0.003

^a^The weights for the three primary indicators are calculated using following methods: The method is Confirmatory Factor Analysis (CFA), where a two-level CFA model is constructed for the aforementioned indicators, with the weight determined as the product of the first-order and second-order path coefficients, denoted as the value.

^b^The data is sourced from the China Education Financial Statistics Yearbook 2024.

### 2.3. Statistical analysis

To assess the impact of selected indicators on the DR policy across provinces, this study employs a spatial regression model. The first step in spatial regression modeling is testing for spatial autocorrelation using global and local Moran index, as shown in formula (1).


$$\left\{ \begin{array}{c} I_{i\text{1}}=\frac{\sum_{i=\text{1}}^{n}\sum_{j=\text{1}}^{n}w_{ij}\left(x_{i}-\overset{―}{x}\right)\left(x_{j}-\overset{―}{x}\right)}{S^{\text{2}}\sum_{i=\text{1}}^{n}\sum_{j=\text{1}}^{n}w_{ij}}\\ {}[4pt]I_{i\text{2}}=\frac{\left(x_{i}-\overset{―}{x}\right)}{S^{\text{2}}}\sum_{j=\text{1}}^{n}w_{ij} \end{array}\;\right.\;$$ (1)

In the equation, x represents the variable with non-random geographic distribution effects. i and j denote the spatial weight matrix based on province longitude and latitude, with w_i,j_ measuring the spatial distance between regions i and j. S^2^ is the sample variance. The upper part of Equation (1) (I_i1_) represents the global Moran index, while the lower part (I_i2_) represents the local Moran I index, testing spatial autocorrelation globally and regionally. Both indices range from −1–1: negative values indicate spatial negative correlation, positive values indicate spatial positive correlation, and values near 0 indicate negligible spatial autocorrelation.

Secondly, this study uses two spatial regression models to identify the impact of selected indicators on DRS, accounting for spatial endogeneity. The first model is the spatial lag model, as shown in Equation (2).


𝐲=𝐠𝐖𝐲+𝐗β+ϵ      (2)

X is the explanatory variable matrix, representing the spatial distribution of indicators across 31 provinces. y is the dependent variable matrix, W is the spatial weight matrix, ɡ is the spatial autoregressive coefficient, β is the parameter matrix, and ε is the random disturbance term. The second model is the spatial errors model, as shown in Equation (3).


$$\left\{ \begin{array}{c} y=X\beta +\mu \\ \mu =\rho W\mu +\varepsilon \end{array}\;\right.\;$$ (3)

where μ is the error term matrix, ρ is the spatial coefficient.

### 2.4. Data source and compliance statement

This study uses Weibo data from 31 provincial-level administrative regions in China, totaling 2,475,833 posts related to the “Double Reduction” policy. The data was collected using web scraping technology and processed through sentiment analysis. All data collection and analysis activities strictly comply with the terms and conditions of the data source and have been approved by the ethics committee. This study does not involve any sensitive personal information, and all Weibo data is publicly accessible. The relevant Ethics Approval Letter and Informed Consent Statement are provided in the “Ethics Documents” section under the “Other” part for the reviewers’ reference.

The study was approved by the Ethics Review Board of Xi’an Jiaotong University which in accordance with the Declaration of Helsinki, and all participants gave written consent to participate in the study.

## 3. Results

### 3.1. Population characteristics and hotspot analysis

Table 2 presents the spatial distribution of DRS and key independent variables across provinces. On average, DRS increased by 21.35% during policy implementation, though substantial regional disparities persist. western and central provinces recorded lower increases of DRS, compared with eastern provinces. Provinces with larger school-age populations reported lower DRS (18.93%) compared to others (22.59%, p < 0.10). Satisfaction was also higher in economically developed regions (25.18%) than in less developed ones (18.60%, p < 0.05).

**Table 2:** Characteristics of the selected sample from 2,475,833 Weibo posts from 31 provincial-level regions in China.

Province	DRS	Pol sup	Mark sup	School sup	θ_1_	θ_2_	θ_3_	θ_4_	θ_5_	θ_6_	θ_7_	θ_8_	θ_9_	θ_10_
Beijing	0.32	1.731	3.768	5.179	3.380	0.061	25	0.441	4.876	0.206	0.049	1.880	0.438	2.145
Tianjin	0.28	1.782	4.254	4.615	3.977	0.028	3	0.247	5.681	0.039	0.066	3.755	0.972	2.393
Hebei	0.23	0.256	0.230	4.067	0.204	0.003	20	0.031	0.299	0.001	0.003	0.201	0.054	0.115
Shanxi	0.2	0.317	0.320	4.045	0.267	0.002	3	0.046	0.414	0.005	0.005	0.150	0.044	0.149
Inner Mongolia	0.22	0.234	0.043	3.790	0.042	0.001	8	0.002	0.058	0.001	0.001	0.019	0.008	0.023
Liaoning	0.19	0.356	0.350	3.313	0.350	0.002	7	0.015	0.469	0.007	0.005	0.259	0.084	0.205
Jilin	0.2	0.491	0.386	3.307	0.385	0.001	3	0.025	0.514	0.005	0.005	0.181	0.063	0.212
Heilongjiang	0.21	0.389	0.169	3.413	0.178	0.001	15	0.007	0.227	0.002	0.002	0.055	0.024	0.095
Shanghai	0.31	3.180	8.097	5.986	7.077	0.071	24	1.361	10.413	0.063	0.127	5.554	0.943	4.160
Jiangsu	0.29	0.292	0.315	5.112	0.282	0.006	12	0.036	0.413	0.002	0.007	0.287	0.076	0.169
Zhejiang	0.28	0.307	0.491	4.950	0.389	0.014	6	0.091	0.625	0.010	0.007	0.293	0.065	0.226
Anhui	0.12	0.251	0.312	3.813	0.281	0.003	3	0.037	0.407	0.004	0.005	0.191	0.062	0.158
Fujian	0.22	0.261	0.226	4.715	0.206	0.007	8	0.027	0.296	0.002	0.005	0.251	0.058	0.123
Jiangxi	0.2	0.328	0.245	3.863	0.239	0.001	2	0.015	0.326	0.004	0.004	0.148	0.058	0.131
Shandong	0.25	0.305	0.242	4.743	0.224	0.004	15	0.024	0.319	0.000	0.005	0.211	0.064	0.135
Henan	0.23	0.265	0.218	4.676	0.196	0.001	21	0.023	0.285	0.004	0.004	0.162	0.043	0.116
Hubei	0.11	0.275	0.214	4.151	0.188	0.002	2	0.019	0.283	0.000	0.003	0.155	0.038	0.113
Hunan	0.2	0.240	0.183	4.154	0.157	0.001	5	0.020	0.240	0.002	0.003	0.122	0.028	0.091
Guangdong	0.28	0.290	0.233	5.001	0.190	0.005	28	0.041	0.296	0.011	0.003	0.152	0.043	0.112
Guangxi	0.21	0.235	0.196	3.985	0.198	0.004	1	0.012	0.261	0.003	0.003	0.152	0.041	0.111
Hainan	0.3	0.836	1.727	3.244	1.647	0.081	1	0.200	2.249	0.055	0.019	0.691	0.236	0.966
Chongqing	0.21	0.382	0.531	4.568	0.512	0.010	2	0.052	0.691	0.030	0.008	0.202	0.073	0.272
Sichuan	0.09	0.201	0.101	4.553	0.093	0.001	3	0.013	0.132	0.001	0.001	0.046	0.018	0.052
Guizhou	0.08	0.352	0.327	3.203	0.310	0.004	1	0.025	0.434	0.002	0.004	0.172	0.050	0.172
Yunnan	0.08	0.209	0.148	3.169	0.143	0.001	1	0.008	0.197	0.003	0.002	0.069	0.020	0.078
Tibet	0.18	0.261	0.094	3.351	0.104	0.000	1	0.006	0.123	0.007	0.001	0.011	0.005	0.057
Shaanxi	0.19	0.306	0.227	3.865	0.210	0.002	2	0.021	0.296	0.014	0.004	0.187	0.035	0.124
Gansu	0.2	0.326	0.196	3.557	0.197	0.001	1	0.004	0.265	0.003	0.002	0.065	0.023	0.110
Qinghai	0.21	0.292	0.114	3.529	0.109	0.002	1	0.003	0.152	0.007	0.001	0.037	0.009	0.061
Ningxia	0.28	0.581	0.983	3.843	0.967	0.008	1	0.044	1.318	0.013	0.009	0.393	0.175	0.543
Xinjiang	0.12	0.197	0.036	3.589	0.038	0.001	1	0.001	0.047	0.004	0.000	0.015	0.007	0.023

### 3.2. Moran index test

Global and local Moran’s I statistics assess the need for spatial econometric modeling (Table 3). The global Moran’s I is 0.019 (p < 0.1), indicating significant spatial clustering of DRS across provinces. Local Moran’s I identifies nine provinces—Beijing, Tianjin, Shanghai, Jiangsu, Zhejiang, Anhui, Sichuan, Guizhou, and Yunnan—with significant intra-regional clustering. These findings confirm non-random spatial dependence in the dependent variable, justifying the use of spatial regression to correct for spatial endogeneity.

**Table 3:** Spatial autocorrelation test.

Variables	Moran index	Variance	P-value	Z-value	Spatial distribution
Panel 1: Global Moran index					
DRS	0.081***	0.036	0.001	3.18	**Spatial clustering**
Panel 2: Local Moran index					
Beijing	0.019***	0.009	0.014	2.198	**Spatial clustering**
Tianjin	0.019***	0.01	0.017	2.121	**Spatial clustering**
Hebei	0.004	0.007	0.213	0.796	Spatial Randomness
Shanxi	−0.001	0.005	0.485	0.038	Spatial Randomness
Inner Mongolia	0.001	0.003	0.281	0.581	Spatial Randomness
Liaoning	−0.001	0.004	0.436	−0.16	Spatial Randomness
Jilin	0.000	0.004	0.457	0.108	Spatial Randomness
Heilongjiang	0.000	0.002	0.393	0.272	Spatial Randomness
Shanghai	0.014***	0.007	0.015	2.174	**Spatial clustering**
Jiangsu	0.009**	0.007	0.072	1.458	**Spatial clustering**
Zhejiang	0.011***	0.007	0.045	1.7	**Spatial clustering**
Anhui	−0.013***	0.005	0.014	−2.189	**Spatial clustering**
Fujian	0.000	0.003	0.337	0.421	Spatial Randomness
Jiangxi	0.000	0.005	0.386	0.289	Spatial Randomness
Shandong	0.005	0.006	0.122	1.166	Spatial Randomness
Henan	0.001	0.004	0.317	0.475	Spatial Randomness
Hubei	−0.003	0.005	0.355	−0.371	Spatial Randomness
Hunan	0.001	0.004	0.344	0.403	Spatial Randomness
Guangdong	−0.001	0.003	0.474	−0.066	Spatial Randomness
Guangxi	0.000	0.003	0.397	0.261	Spatial Randomness
Hainan	−0.003	0.003	0.26	−0.643	Spatial Randomness
Chongqing	0.000	0.004	0.406	0.237	Spatial Randomness
Sichuan	0.005***	0.004	0.043	1.713	**Spatial clustering**
Guizhou	0.007***	0.004	0.016	2.138	**Spatial clustering**
Yunnan	0.007***	0.003	0.001	3.068	**Spatial clustering**
Tibet	0.001	0.001	0.117	1.192	Spatial Randomness
Shaanxi	0.000	0.003	0.354	0.375	Spatial Randomness
Gansu	0.000	0.004	0.394	0.268	Spatial Randomness
Qinghai	0.000	0.002	0.354	0.374	Spatial Randomness
Ningxia	−0.002	0.004	0.353	−0.376	Spatial Randomness
Xinjiang	0.000	0.001	0.339	0.416	Spatial Randomness

^a^The spatial relationship uses the “contiguity edges corners” type (CONTIGUITY_EDGES_CORNERS), and the distance is based on the “Euclidean distance” (Euclidean Distance). *, **, and *** indicate statistical significance at the 5%, 1%, and 0.1% levels, respectively.

### 3.3. Spatial regression models

Building on the preceding analysis, this study employs spatial regression models to examine the associations between selected indicators and DRS (Table 4). Model 1, incorporating all secondary indicators, reveals that central fiscal support, local fiscal support, marketing education revenue, the number of marketing institutions, and education employment in the market sector are positively associated with DRS. Sport resource density also exerts a positive effect, whereas student–teacher ratio and information support are negatively associated with DRS.

**Table 4:** Spatial regression results of selected indicators on DRS^a^.

Variables	Model 1	Model 2	Model 3	Model 4	Model 5
θ_1_	0.051*** (0.016)				
θ_2_	0.015*** (0.004)				
θ_3_	0.011 (0.009)				
θ_4_	0.021*** (0.008)				
θ_5_	0.090*** (0.039)				
θ_6_	0.010* (0.006)				
θ_7_	−0.033* (0.02)				
θ_8_	0.003 (0.013)				
θ_9_	0.051*** (0.016)				
θ_10_	−0.003*** (<0.001)				
Political support		0.021*** (0.009)	0.015 (0.012)	0.017 (0.012)	0.020 (0.009)
Market support		0.004 (0.009)	−0.004 (0.014)	0.004 (0.009)	−0.004 (0.011)
School support		0.022*** (0.006)	0.023*** (0.006)	0.017 (0.011)	0.010 (0.012)
Market support*Political support			0.004 (0.004)		
School support*Market support				0.002 (0.004)	
School support*Political support					0.005 (0.004)
Constant	0.067*** (0.026)	0.112*** (0.019)	0.119*** (0.021)	0.122*** (0.026)	0.132*** (0.025)
R^2^	0.323	0.168	0.175	0.174	0.171

^a^*, **, and *** indicate statistical significance at the 5%, 1%, and 0.1% levels, respectively.

Model 2 aggregates the indicators into three dimensions—market, political, and educational support—and demonstrates that each exerts a positive effect on DRS. Models 3 through 5 test interaction effects among the three supports. Results indicate significant positive moderating effects between market and political support, market and educational support, and political and educational support.

### 3.4. Subgroup analysis

**Fig 1:**
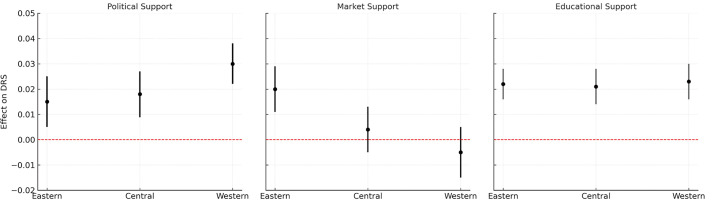
Subgroup analysis of three support on DRS.

A subgroup analysis investigates regional heterogeneity in the effects of political, market, and educational support on DRS. As illustrated in Fig 1, political support exerts a stronger positive influence in western provinces, market support demonstrates greater efficacy in eastern regions, while educational support shows no significant variation across provinces.

## 4. Discussion

This study assesses the implementation effects of China’s Double Reduction (DR) policy through sentiment analysis and spatial regression modeling. While public satisfaction with the policy is generally positive, substantial regional disparities persist, particularly between more and less developed provinces. Political, market, and educational support each exert a significant positive influence on DR policy satisfaction (DRS), underscoring the effectiveness of government-led educational reforms.

### 4.1. Regional disparities in satisfaction with the double reduction policy

This study reveals significant regional disparities in public satisfaction with the DR policy. Provinces in eastern and central China report higher satisfaction, whereas western and densely populated regions consistently show lower levels. These findings align with evaluations of other national education initiatives, such as rural revitalization programs and universal preschool services [15,16]. Such spatial patterns likely reflect more advanced economic development and greater sociocultural openness in eastern provinces. Overall, the broadly positive distribution of DRS underscores the Chinese government’s commitment to policy implementation.

### 4.2. The positive effect of political support

We further examine the effects of the three types of support. First, political support exerts a significant positive influence on DRS. Both central and local fiscal allocations are positively and significantly associated with satisfaction levels. Political support plays a critical role in the implementation of education policy not only in socialist contexts such as China but also in capitalist systems like the United States and Japan [17–21] This influence stems from the fact that political endorsement often ties policy enforcement to local government performance, thereby enhancing administrative commitment—a mechanism well documented in prior studies [20,21].

### 4.3. The positive effect of marketing support

Second, market support exerts a significant positive effect on DRS. All three indicators—marketing education revenue, the number of private institutions, and employment in the private education sector—are significantly associated with higher satisfaction levels. Prior studies indicate that market support is particularly salient in shaping public attitudes toward the DR policy, given its unique focus [22,23]. As a reform aimed at compulsory education, the DR policy seeks to reduce academic pressure on students and parents. In China, a substantial portion of this burden originates from dependence on private tutoring services. Consequently, regulating and supporting the private education market is critical to effective policy implementation.

### 4.4. The positive effect of educational support

Third, educational support demonstrates mixed effects on DRS. Sport Resource Density is positively associated with satisfaction, consistent with the DR policy’s goal of reducing academic pressure through physical activity. In contrast, Student–Teacher Ratio and ICT Resource Density show negative associations. The inverse relationship with Student–Teacher Ratio reflects that higher values indicate fewer teachers per student, potentially weakening policy implementation. The negative effect of ICT Resource Density may indicate unintended outcomes, as existing research suggests that extensive use of digital tools may elevate student stress [24].

### 4.5. Moderation by political, market, and educational support

Our findings confirm that the three types of support—political, market, and educational—interact positively to moderate the effects on DR policy satisfaction. This suggests a synergistic relationship among these supports, forming a virtuous coupling that jointly facilitates effective policy implementation. The positive results also validate the feasibility of the proposed “government–market–school” analytical framework. In practice, the successful implementation of education reforms requires the coordinated engagement of multiple stakeholders and a comprehensive evaluation approach [13,18–20].

### 4.6. Regional heterogeneity

We conclude by examining the regional heterogeneity in the effects of the three types of support. Results indicate that political support has a stronger positive influence on DRS in western provinces, while market support exerts greater effects in eastern provinces. No significant interprovincial variation emerges for educational support. These patterns align with China’s spatial-cultural context: eastern provinces, characterized by more advanced economies and open social structures, respond more to market-oriented support; in contrast, central and western regions—closer to political centers and less economically developed—depend more on political support [25].

### 4.7. Policy recommendations

To enhance policy implementation, the government should introduce targeted measures that promote equitable access to compulsory education, establishing an integrated DR policy framework analogous to France’s “Priority Education Zones” [25] and Japan’s “Integrated Primary–Secondary System” [26].” Schools should improve teacher quality through collaborative professional networks and strengthen governance structures in underperforming institutions to ensure regional parity.

To reduce regional disparities, targeted interventions are required. In economically disadvantaged central and western provinces, the government should enhance fiscal allocation mechanisms, implement incentive schemes to attract and retain qualified teachers, and establish inter-provincial teacher mobility programs to equalize educational resources. In more developed eastern regions, strategic school planning is essential to alleviate overcrowding and address structural inequities in educational access.

Advance the shift toward quality education. Teacher deployment strategies should prioritize instructional quality, emphasizing professional competence and pedagogical effectiveness. Curriculum reform must promote holistic development by integrating moral, intellectual, physical, aesthetic, and labor education. Additionally, strengthening vocational education through robust school–industry partnerships is vital for addressing educational disparities and aligning learning outcomes with labor market needs.

### 4.8. Strengths and limitations

This study provides a comprehensive examination of the determinants of public satisfaction with China’s DR policy by employing sentiment analysis and spatial econometric modeling. The use of a large-scale, regionally diverse dataset enhances the robustness of the findings and offers critical insights into spatial and resource-related factors shaping policy reception. By incorporating political, market, and educational support dimensions, the study contributes to a more nuanced understanding of the multifaceted dynamics involved in education policy implementation.

Nonetheless, several limitations warrant consideration. Despite efforts to control for major confounding variables, unobserved heterogeneity—particularly in regions with complex socio-economic structures—may still bias the results. The exclusive focus on mainland China constrains the external validity of the findings for contexts with differing institutional and educational frameworks. Moreover, the endogenous allocation of policy resources may pose challenges for causal inference. Future research should employ longitudinal or quasi-experimental designs to improve causal identification and generalizability.

## 5. Conclusions

This study finds generally high public satisfaction with the DR policy, though substantial regional disparities persist. DRS is positively associated with political, market, and educational support, highlighting the critical role of regional conditions and resource allocation in shaping policy outcomes. Future research should investigate the long-term impacts of such reforms across diverse socio-economic settings.
